# Pediatric autoinflammatory bone disorders—a mini review with special focus on pathogenesis and inborn errors of immunity

**DOI:** 10.3389/fped.2023.1169659

**Published:** 2023-06-05

**Authors:** Rebecca Hetrick, Melissa Oliver

**Affiliations:** Division of Pediatric Rheumatology, Indiana University School of Medicine, Indianapolis, IN, United States

**Keywords:** autoinflammatory bone disorders, chronic nonbacterial osteomyelitis, chronic recurrent multifocal osteomyelitis, synovitis acne pustulosis hyperostosis osteitis (SAPHO) syndrome, Majeed syndrome, deficiency of the interleukin-1 receptor antagonist

## Abstract

Autoinflammatory bone disorders are a group of diseases characterized by sterile osteomyelitis. This includes chronic nonbacterial osteomyelitis and the monogenic forms, Majeed syndrome and deficiency of the interleukin-1 receptor antagonist. These disorders result from innate immune system dysregulation and cytokine imbalance that triggers inflammasome activation causing downstream osteoclastogenesis and excessive bone remodeling. In this review, we will summarize the immunopathogenesis of pediatric autoinflammatory bone diseases with a special focus on the genetics and inborn errors of immunity, while briefly touching on the clinical manifestations and management of each disease as well as areas for future research.

## Introduction

Autoinflammatory bone disorders are characterized by sterile bone inflammation that results from innate immune system dysregulation and cytokine imbalance, leading to NLRP3 inflammasome overactivation. This results in pathogenic osteoclastogenesis with excessive bone turnover and bone formation. This review will focus on the genetic mechanisms and inborn errors of immunity that drive sterile bone inflammation in these disorders. These mechanisms provide a framework for understanding the clinical manifestations and management of each disease, as well as potential areas for future research.

### Nomenclature

Autoinflammatory bone disorders (ABDs) can be classified as sporadic or monogenic. Sporadic forms of the disease were previously known as chronic recurrent multifocal osteomyelitis (CRMO). However, with increased recognition of unifocal and non-recurrent presentations, the broader term chronic nonbacterial osteomyelitis (CNO) is preferred. Synovitis, Acne, Pustulosis, Hyperostosis, and Osteitis (SAPHO) syndrome also falls within the category of sporadic ABDs. SAPHO tends to present in adults and may represent a part of the CNO spectrum or represent a unique entity within the adult population (1, 2). Majeed syndrome and deficiency of the interleukin-1 receptor antagonist (DIRA) are monogenic ABDs that result from homozygous mutations in the disease-associated gene. Pyogenic arthritis, pyoderma gangrenosum and acne (PAPA), a monogenic autoinflammatory syndrome that can present with sterile bone inflammation, will be briefly discussed (3).

### Epidemiology

Despite increased awareness of CNO over the last decade, the disease remains relatively rare. Surveillance data from 2006 to 2008 in Germany estimated an annual incidence of 0.45 per 100,000 children but more recent data suggest a higher incidence (4, 5). Aden et al. demonstrated an increase in the incidence of CNO cases from 8 per million children from 2005 to 2015 compared to 23 per million children from 2016 to 2019 (6).

CNO is typically diagnosed in children and adolescents but can occur in adulthood. In contrast, SAPHO syndrome presents more in adults but can also manifest in children and adolescents. CNO appears more prevalent in females than males with an average age of disease onset occurring between 9 and 11 years of age (6–14). DIRA, Majeed and PAPA are exceptionally rare with a handful of case reports and case series available. DIRA presents at birth or within the first few weeks of life, though intrauterine onset has been described (15, 16). Patients with Majeed present in early childhood most commonly before the age of 2 years (17). PAPA can present in early to late childhood with adult onset also described (18, 19).

## Pathogenesis and genetics

### Overview

By definition, autoinflammatory diseases are characterized by increased systemic inflammation and an absence of autoantibodies or antigen-specific T cells. In ABDs, research shows that cytokine imbalance and innate immune system dysregulation lead to impaired osteoclastogenesis with increased osteoclastic activation and bone remodeling. This causes the development of sterile bone inflammation. through aberrant activation of the NLRP3 inflammasome, which is an intracellular multi-protein complex involved in host innate immune defenses. In response to microbial infection and/or cellular damage, the NLRP3 inflammasome becomes activated, ultimately leading to secretion of pro-inflammatory cytokines, primarily IL-1 β, which stimulates osteoclast formation (20, 21).

The pathogenesis of CNO is likely multifactorial. An infectious trigger has been investigated but a clear link has yet to be identified. Bone cultures growing microorganisms *Propionibacterium acnes, Mycoplasma* and *Staphylococcus aureus* and a partial disease response to azithromycin has been reported (22–29). However, Girschick et al. evaluated this association and found no evidence of bacterial infection in bone biopsy of pediatric CNO patients, suggesting that positive microbial cultures may represent contaminants (23). Whether or not a preceding infection or microbial exposure is involved in the onset of immune dysregulation requires further exploration. Nevertheless, most experts agree that antibiotics alone are insufficient to manage CNO (7, 13, 23, 30).

Inborn errors of immunity and other genetics likely also contribute to the pathogenesis of CNO. Mutations within the NLRP3 inflammasome are associated with ABDs, with specific mutations involving the interleukin (IL)-1 receptor antagonist gene and lipin-2 gene implicated in the monogenic forms of CNO (21, 31). These mutations lead to increased production of the cytokine IL-1β and other pro-inflammatory mediators, which are involved in the pathogenesis of sterile bone inflammation (15). We will further explore the genetics and immunopathogenesis involved in ABDs (summarized in Table 1).

**Table 1:** Summary of ABDs, associated genetic mutation, and impact on immune function.

ABD	Gene	Normal role in immune system pathways	Mutation effect in associated ABD	Mutation impact on immune function
DIRA	*IL1RN*	- Regulation of IL-1 secretion	Absence of IL-1Ra expression or expression of a non-functional protein.	- Impaired control of IL-1 signaling - Unopposed IL-1R activation IL-1α and IL-1β stimulation - Activation of the NLRP3 inflammasome
Majeed	*LPIN2*	- Lipid metabolism - Regulation of gene transcription - Controlling inflammation - Inhibition of P2X7R	Loss of function mutation in lipin-2 gene	- Increased proinflammatory cytokine, e.g., IL-1β - Increased P2X7R activation - Activation of the NLRP3 inflammasome
PAPA	*PSTPIP1*	- Immunity - Cytoskeleton regulation	Increased binding with pyrin	- Impaired inhibition of the NLRP3 inflammasome - Increased IL-1β production
Sporadic CNO	Unknown, Multifactorial	n/a	n/a	- Impaired signaling of the MAP kinase to ERK 1/2 pathway - Increased production of pro-inflammatory cytokines - Decreased the phosphorylation of the IL-10 promoter region - Activation of the NLRP3 inflammasome

### Monogenic CNO

#### IL1RN

Cytokine IL-1 is a key mediator of the innate immune system's inflammatory response (21, 31). IL-1 exists in two forms, IL-1α and IL-1β, both of which are pro-inflammatory cytokines. IL-1 signals through the IL-1 receptor (IL-1R) and IL-1R antagonist (IL-1Ra) regulates IL-1 activity by blocking binding of IL-1 to IL-1R. IL-1β secretion occurs via activated caspase-1, the IL-1 converting enzyme, within the multi-protein complex NLRP3 inflammasome. Within the inflammasome, caspase-1 cleaves pro-interleukin-1β to the biologically active form, IL-1β (32–34). IL-1β induces the expression of genes involved in the acute inflammatory response and fever control. Overproduction of IL-1β has been implicated as the driving force in the pathogenesis of many autoinflammatory disorders (21, 31). Mutations in the IL-1Ra gene (*IL1RN*) can either cause an absence of IL-1Ra expression or expression of a non-functional protein. This results in impaired control of IL-1 signaling, unopposed IL-1R activation and IL-1α and IL-1β hyperresponsiveness and aberrant inflammatory response (15). Patients with DIRA, one of the monogenic forms of CNO, have mutations in *IL1RN* (15, 35).

#### LPIN2

Lipin-2 gene *(LPIN2)* encodes the protein called lipin-2. Lipin proteins have important roles in lipid metabolism and gene expression as transcriptional co-regulators in adipogenesis. They have also been implicated in controlling inflammation through maintenance of cellular membrane cholesterol levels and adipocyte inflammatory gene expression. *LPIN2* is found in the liver, intestines and white blood cells (36). *LPIN2* is a phosphatidate phosphatase (PAPs) and loss of PAP activity causes increased inflammation (36, 37). Mutations in *LPIN2* lead to cytokine imbalances and deficiency of *LPIN2* creates an increase in pro-inflammatory cytokines (IL-1β, IL-6, IFN-*γ* and TNFα). With reduced or deficient lipin-2 expression in macrophages, increased activation of the NLRP3 inflammasome triggered by the innate immune response results in IL-1β overproduction (36, 38). Lorden et al. has also described lipin-2 as a negative innate immune system regulator through its interaction with the P2X7 receptor (P2X7R) (37). P2X7R is expressed by most immune cells and induces a variety of cellular response, including inflammation, cell proliferation and death, and phagocytosis. Disruptions in the binding of this receptor on macrophages trigger inflammasome activation. *LPIN2* may inhibit the activation and sensitization of the P2X7R. A deficiency of lipin-2 protein allows for increased activation of this receptor which downstream activates the NLRP3 inflammasome (37).

A loss of function mutation in *LPIN2* has been found in affected individuals with Majeed Syndrome, another rare monogenic form of CNO. Five different mutations in the *LPIN2* gene have been reported in consanguineous families. Most patients are homozygous for a nonsense or splice mutation in *LPIN2* gene, although one mutation is reported as a missense mutation (Ser734Leu) (17). While the link between lipin-2 and inflammasome regulation has been established, further research is needed to understand the preferential target on bone that produces the clinical phenotype in Majeed Syndrome.

### Other genetic associations

Sporadic CNO lacks a direct genetic association. However, a genetic predisposition may in the risk of developing sterile bone inflammation when combined with other factors.

The Pombe Cdc15 homology (PCH) family is a group of proteins involved in immunity and cytoskeleton regulation (39). Proline-serine-threonine phosphatase-interacting protein (*PSTPIP)1* and *PSTPIP2* proteins are part of the PCH family and are preferentially expressed in the hematopoietic system. Mutations in these proteins have been associated with autoinflammatory disorders (39, 40). Mutations in *PSTPIP1* cause pyogenic arthritis, pyoderma gangrenosum, and acne (PAPA) syndrome in humans, while an autosomal recessive mutation in *PSTPIP2* has been implicated in the development of multifocal osteomyelitis in mouse models (the *cmo* mouse) (39–41).

*PSTPIP1* mutations can lead to increased binding with pyrin, a protein produced on monocytes involved in the apoptotic and inflammatory signaling pathways. This enhanced interaction of pyrin and *PSTPIP1* results in impaired inhibition of the inflammasome and increased IL-1β production in peripheral mononuclear cells (39, 42). *PSTPIP1* mutations specifically in T lymphocytes attenuate the phosphorylation of extracellular signal regulated kinase (ERK)1 and ERK2, which are mitogen-activated protein kinases involved in intracellular signal transduction and priming the inflammasome through lipopolysaccharide activation (43, 44). Impairments in the ERK1/2 pathway have also been implicated in the pathogenesis of sporadic CNO, which is discussed later in this review (45, 46).

The *PSTPIP2* gene is located on chromosome 18 in both mice and humans and is selectively expressed in macrophages. Disruption of the *PSTPIP2* protein creates excessive macrophage accumulation and increased production of pro-inflammatory mediators, such as chemokine macrophage inflammatory protein-1α (MIP-1α) and cytokine IL-6 (48). The loss of regulation of macrophages and inflammatory mediators promotes osteoclast and osteoblast activation (49). A missense mutation, specifically L98P, has been found to alter the structure and/or function of *PSTPIP2* causing sterile bone inflammation in mice that mimics CNO seen in humans (41, 47, 48). The paws of the *cmo* mouse have elevated levels of pro-inflammatory cytokines and chemokines, particularly IL-1β. The *cmo* mouse will then develop sterile osteomyelitis in their tails and paws beginning at 4 to 6 weeks (41). While this mutation in *PSTPIP2* is implicated in the disease pathogenesis of CNO in mice, its role in human CNO is unclear.

*FBLIM1* is another gene where mutations have been implicated in the development of sterile bone inflammation. This encodes filamin-binding LIM protein 1 (FBLP-1), which is involved in bone remodeling through the regulation of receptor activator of nuclear factor (NF)-kβ (RANK) ligand (RANKL) activation via the ERK1/2 phosphorylation pathway mentioned earlier with mutations involving *PSTPIP1*. RANK/RANKL signaling controls normal osteoclast function and is important for bone homeostasis (50, 51). RANKL binds to its receptor RANK on osteoclast precursors resulting in the activation of multiple signaling pathways that lead to osteoclast differentiation and increased bone turnover **(**50)*.* Using whole exome sequencing, Cox et al, identified mutations in the *FBLIM1* gene in two children with CNO and psoriasis. It is postulated that when FBLP1 is absent or dysfunctional, this leads to increased ERK1/2 phosphorylation and increased RANKL production that causes osteoclast activation, bone resorption, and inflammasome activation (52).

Further supporting a possible genetic susceptibility for CNO, Golla et al. genotyped CNO patients and their parents and found a significant association with a rare allele of marker D18S60 on chromosome 18q21.3–22 (53). Another researcher, Charras, et al., performed whole exome sequencing in families with CNO and found rare damaging heterozygous variants within the P2X7R sequence, which may play a role in CNO development and severity of the disease (54). Both need further investigations, but these suggest there may be other genes that could predispose individuals to develop CNO.

### Sporadic CNO

Although lacking a single responsible gene, the sterile bone inflammation in sporadic CNO involves many of the same pathways and cellular machinery as the monogenic forms. It develops from a combination of impaired innate immune responses and an imbalance of cytokine expression (21, 55). Monocytes from sporadic CNO patients fail to express the immune regulatory cytokines IL-10 and IL-19 because of impaired signaling of the MAP kinase (MAPK) to theERK 1/2 phosphorylation pathway. Other environmental stress- or mitogen response-signaling pathways, the c-Jun N-terminal kinase (JNK) and p38 MAPK pathways, are unaffected. Thus, this imbalance leads to increased production of pro-inflammatory cytokines (IL-1β, IL-6, TNFα, monocyte chemoattractant protein 1, MIP1-β) (45, 46). Additionally, there is a decreased phosphorylation of the IL-10 promoter region due to genetic polymorphisms resulting in further impaired the regulatory cytokine IL-10 expression. Of note, this reduced IL-10 expression may be related to *FBLIM1*'s involvement in disease pathogenesis mentioned earlier because IL-10 mediates transcription factor Signal Transducer and Activator of Transcription (STAT)3 which increases the gene expression of *FBLIM1* (45). Recognition of intracellular danger signals (PRR, TLR, NLR) by CNO monocytes further amplifies the NLRP3 inflammasome activity (33). Ultimately, the increased activation of NLRP3 inflammasome causes increased production of IL-1β which leads to pathogenic osteoclastogenesis via enhanced RANK/RANKL interaction on osteoclast precursor cells, inducing osteoclast differentiation and activation and excessive bone turnover and bone formation (56, 57).

## Clinical presentation

### DIRA

DIRA syndrome (OMIM #612852) is an autosomal recessive disorder characterized by neonatal onset of systemic inflammation, sterile bone inflammation, and pustular skin lesions. Many neonates present to medical attention with fevers and a septic-like picture that mimics serious bacterial infections (15, 58). However, DIRA represents a clinical spectrum and later presentations have been described (59, 60). Other common findings in DIRA include oral mucosal lesions, nail changes, conjunctival injection, and hepatosplenomegaly (15). If unrecognized and untreated, DIRA carries a significant risk of mortality (58). Delays in treatment can also lead to severe sequelae such as failure to thrive, skeletal deformities, and interstitial lung disease (15, 58).

### Majeed

Majeed syndrome (OMIM #609628) is an autosomal recessive disorder first described in 3 related children presenting with sterile bone inflammation and congenital dyserythropoietic anemia (CDA) (61). Since these first cases, 21 additional affected individuals have been described. Sterile bone inflammation and microcytic dyserythropoietic anemia are the most prominent clinical features, occurring in 91% of affected individuals. Skin manifestations, primarily neutrophilic dermatoses, are considered characteristic of the disease but only appear in 14% (62). Other common findings include elevated inflammatory markers (88%), recurrent fevers (46%), failure to thrive (38%), hepatosplenomegaly (30%), and neutropenia (13%) (17).

### PAPA

Pyogenic Arthritis, Pyoderma Gangrenosum, and Acne syndrome (OMIM **#**604416) is an autosomal dominant disorder characterized by aseptic arthritis, pyoderma gangrenosum, and cystic acne. However, only 25% of patients experience the triad of manifestations. Arthritis occurs in most patients and can be erosive, polyarticular or oligoarticular, and typically affects peripheral joints (63). Cystic acne affects an estimated 10%–20%, and fevers accompany disease flares in 27% (18, 19, 63). Sterile osteomyelitis is reported in some cases of PAPA with one series finding sterile bone inflammation in 8% (3, 63, 64).

### Sporadic CNO

CNO typically presents with bone pain with or without associated swelling or warmth. CNO is a diagnosis of exclusion without validated diagnostic criteria, and ruling out mimicker diseases, such as infection and malignancy, plays a role in the evaluation. CNO lesions can affect any bone but most commonly occur in the metaphyseal regions of long bones. However, epiphyseal and diaphyseal lesions can be seen, occurring in 37% of patients in one case series (9). Other commonly affected bones include the mandible, clavicles, vertebrae, and bones of the pelvis. Bone involvement can be unifocal or multifocal and symmetric or asymmetric (6–14, 64).

Many CNO patients experience inflammation elsewhere in the body, most notably in the joints, skin, and gut (6–14). Joint involvement may be seen in up to 40% of CNO patients and is typically monoarticular or oligoarticular, but polyarticular disease can occur (9, 10). Likewise, arthritis in CNO prefers joints adjacent to boney lesions but can also occur at distant sites (10, 13). Common associated skin conditions include psoriasis, palmoplantar pustulosis, and severe acne, occurring in anywhere from 10%–24% of CNO patients. Comorbid inflammatory bowel disease is estimated to occur in 3%–8% of CNO patients (6–14).

SAPHO presents with similar bone pain and skeletal findings as CNO except there is a predilection for the sternoclavicular area. Patients with SAPHO have more cutaneous involvement, particularly palmoplantar pustulosis(66).

## Diagnosis

### Laboratory evaluation

There are no specific lab findings or pathognomonic autoantibodies for ABDs (21, 67). Monogenic ABDs typically have evidence of systemic inflammation with elevated c-reactive protein (CRP) and erythrocyte sedimentary rate (ESR) and/or leukocytosis (15, 17–19). In sporadic CNO, lab evidence of systemic inflammation may or may not be present (6–14).

### Imaging evaluation

Radiologic evaluation plays a critical role in detecting osteitis and evaluating disease extent. Plain radiographs are generally first-line for the evaluation of bone pain in children. However, they have low sensitivity for early disease findings. MRI has emerged as the gold standard for the evaluation of osteitis (68, 69). MRI can demonstrate early disease findings such as bony edema or altered diffusion capacity on diffusion-weighted imaging. Other findings of active lesions include periosteal reaction, hyperostosis, and surrounding soft tissue inflammation (65). MRI can also reveal disease morbidities such as pathologic fractures, vertebral compression fractures, or growth plate disruption (70). Whole-body MRI is preferred given the presence of clinically silent lesions in up to 29% of patients (12, 69). While bone scintigraphy may be used when whole-body MRI is unavailable, this modality is less sensitive and involves radiation exposure (9, 68).

### Bone biopsy

Bone biopsy remains an important diagnostic tool in certain cases. Clinicians may opt to forgo bone biopsy in patients with a typical clinical presentation and compatible imaging findings. However, certain clinical scenarios, such as the presence of solitary lesions, cytopenia, or excessively elevated inflammatory markers, may warrant biopsy if infection or malignancy remains on the differential (71). Histopathology of bone samples can vary depending on the stage of the disease. Early lesions are characterized by the presence of neutrophils and monocytes (72, 73). Older lesions are characterized by the presence of lymphocytes and plasma cells and often demonstrate chronic inflammatory change with some degree of fibrosis or sclerosis (7, 72, 73).

## Management of autoinflammatory bone diseases

Treatment of monogenic ABDs appears to correlate with the understood pathophysiology of these syndromes. Both anakinra, a recombinant human IL-1Ra, and rilonacept, a recombinant fusion protein that binds to both IL-1β and IL-1apha, are recommended first-line agents for DIRA (74, 75). IL-1β antagonist canakinumab does not appear as consistently efficacious as these agents, possibly due to ongoing IL-1α activity in these patients (60, 75, 76). Similarly, Majeed Syndrome appears to response well to anti-IL-1 activity, with reports of efficacy with anakinra and canakinumab (77, 78). However, the management of PAPA is much more variable. Good response has been reported to corticosteroids, anakinra, canakinumab, TNF inhibitors, and tocilizumab (63).

Therapies for sporadic CNO either mediate pro-inflammatory cytokine activity or target osteoclast activity. Treatment of sporadic CNO has advanced in recent years with the development of the Childhood Arthritis and Rheumatology Research Alliance (CARRA) consensus treatment plan (68). First line therapy for CNO patients is typically non-steroidal anti-inflammatory drug (NSAID) therapy unless spine involvement is present. NSAIDs inhibit the activity of cyclooxygenases (COX). These enzymes convert arachidonic acid into prostaglandins, which are required for osteoclast activation (56, 79).

Unfortunately, only about 40% of sporadic CNO patients achieve disease control with NSAIDs monotherapy. Many require escalation to biologic or non-biologic disease-modifying antirheumatic drug (DMARD) therapy and/or bisphosphonates (9, 14). Non-biologic DMARDs, such as methotrexate and sulfasalazine, provide immunomodulatory effects through multiple mechanisms, but the direct therapeutic mechanism of these agents in sporadic CNO remains unclear (79). Biologic DMARDs function by disabling or blocking the activity of specific pro-inflammatory cytokines. TNF-α inhibitors are the most common biologic DMARDs used in sporadic CNO. These medications inactivate TNF-α, a very potent pro-inflammatory cytokine found to be elevated in the monocytes and serum of patients with sporadic CNO (45, 56). Bisphosphonates are preferred in patients with spinal lesions and often used in patients with NSAID-refractory disease (68). Bisphosphonates work through a variety of mechanisms to inhibit osteoclast activity and impair osteoclast function, but the exact therapeutic mechanism in sporadic CNO remains unclear (79).

Lastly, given the implication of the IL-1 pathway in the pathogenesis of CNO, modulation of this pathway has been proposed as a promising therapy. However, treatment of sporadic CNO with anti-IL-1 therapy has shown mixed results (80–82).

## Future directions

Despite the major advances made over the last twenty years, there are still many unanswered questions in ABDs research. CNO remains a diagnosis of exclusion. With no specific biomarkers, patients often experience diagnostic and treatment delays. A group of international experts and CNO patients and families are working to develop the first classification criteria for pediatric CNO to aid in the earlier identification of these patients (83, 84).

Unfortunately, most data on ABDs comes from case reports and series or retrospective cohort studies. The CRMO/CNO workgroup within CARRA has established an international prospective disease registry (CHronic nonbacterial Osteomyelitis International Registry (CHOIR)) for patients with chronic nonbacterial osteomyelitis (CNO). From the CHOIR registry, a prospective observation study is currently underway to determine the effectiveness of the consensus treatment plans previously developed by the CARRA workgroup. The registry will aid researchers in conducting comparative effectiveness trials (85). At this time, the field of ABDs lacks validated outcomes measures that would facilitate comparative effectiveness of different therapies. The OMERACT (Outcome Measures in Rheumatoid Arthritis Clinical Trials) CNO/SAPHO working group was established in 2019 to develop a core outcome measurement set through a data driven and consensus process for CNO and SAPHO for all clinical trials (86).

## Conclusion

Autoinflammatory bone disorders constitute a heterogeneous group of rare diseases with both sporadic and monogenic forms. Extraosseous manifestations, most commonly involving the skin, often occur. Dysregulation of the innate immune system and cytokine imbalance contribute to disease pathogenesis. Critical areas of future research include the development of diagnostic and classification criteria, enrollment in large prospective cohorts, and creation of standardized outcomes measures.
